# Oral Hygiene Status in Children on the Autism Spectrum Disorder

**DOI:** 10.3390/jcm14061868

**Published:** 2025-03-10

**Authors:** Magdalena Prynda, Agnieszka Anna Pawlik, Ewa Emich-Widera, Beata Kazek, Mikołaj Mazur, Wojciech Niemczyk, Rafał Wiench

**Affiliations:** 1 Orthodontic Specialist, M-Dent Center for Esthetic Dentistry and Implantology, 34a/7 Sienkiewicza St., 50-335 Wrocław, Poland; 2 Specialist Dental Clinic dr n.med. Agnieszka Anna Pawlik, ul.Strumieńskiego 12/4, 41-400 Mysłowice, Poland; 3 Department of Child Neurology, Faculty of Medical Sciences, Medical University of Silesia, 40-752 Katowice, Poland; 4 Development Assistance Centre “CWR Persevere”, Kępowa 56 st., 40-583 Katowice, Poland; 5 Faculty of Medical Sciences in Zabrze, Medical University of Silesia, Pl. Traugutta 2, 41-800 Zabrze, Poland; 6 Department of Periodontal Diseases and Oral Mucosa Diseases, Faculty of Medical Sciences in Zabrze, Medical University of Silesia, Pl. Traugutta 2, 41-800 Zabrze, Poland

**Keywords:** autistic disorder, children, dental care, dental caries, dentistry, oral health, oral hygiene

## Abstract

**Background/Objectives:** Children with autism spectrum disorder (ASD) often face challenges in maintaining oral hygiene due to sensory sensitivities, behavioral difficulties, and limited access to specialized dental care. This study aimed to assess the oral hygiene status of children with ASD and compare it with neurotypical peers. **Methods**: A cross-sectional study was conducted with 74 children with ASD and 74 neurotypical children. Dental exams measured oral hygiene and caries prevalence using the DMFT/dmft, Oral Hygiene Index (OHI), and Sulcus Bleeding Index (SBI). Tooth brushing frequency and dental visits were also recorded. Statistical analysis was performed using the Mann–Whitney U test and Fisher’s exact test. **Results**: Children with ASD had significantly poorer oral hygiene and higher caries rates compared to controls. Boys with ASD had higher DMFT scores, indicating more caries. Additionally, ASD children brushed their teeth less often and had fewer dental visits. Preventive treatments were underutilized in this group despite a higher need. **Conclusions**: Children with ASD face notable oral health challenges, including poor hygiene, higher caries prevalence, and limited preventive care. These findings highlight the need for tailored interventions, improved parental education, and specialized dental care strategies for this population.

## 1. Introduction

Autism spectrum disorder (ASD), also known as autism, is a prevalent neurodevelopment disorder that is strongly influenced by genetic factors and manifests in a variety of ways. It is characterized by underlying cognitive features and frequently co-occurs with other conditions. The core symptom domains of autism can thus be classified into two categories: social communication impairments and restrictive, repetitive behavioral patterns and interests [1,2,3]. ASD is regarded as a disorder that exhibits a spectrum of presentations, given that its manifestations vary among individuals [4]. The diagnosis of ASD is typically made in children aged two years, although it can be diagnosed in children at a later age, depending on the complexity of symptoms and the severity of the disorder [5,6]. Despite the absence of a direct impact of autism on oral cavity health, the inability of patients to adhere to proper oral hygiene practices, and their concomitant challenges in accessing dental care and therapeutic interventions, leaves them susceptible to an elevated risk of developing carious lesions, periodontal disease, alterations in oral microbiota, and an augmented probability of traumatic injury [7,8,9]. A notable challenge in providing dental care for individuals with ASD is their resistance to routine oral hygiene practices when these are implemented by caregivers. Moreover, there is a paucity of professionals specializing in the provision of dental treatment for individuals with ASD [10,11,12]. Dental caries and erosions are multifactorial conditions influenced by a combination of genetic, dietary, and nutritional factors. Genetic susceptibility plays a role in enamel composition, saliva production, and microbial colonization, which can increase susceptibility to caries. Dietary habits, particularly high consumption of fermentable carbohydrates, sugary snacks, and acidic beverages, contribute significantly to enamel demineralization and caries progression. Nutritional deficiencies, such as low calcium, vitamin D, and phosphate levels, can impair enamel remineralization, increasing the risk of erosion and decay. Studies have shown that children with ASD often have restricted diets and selective eating habits, which may exacerbate oral health problems due to deficiencies in essential nutrients and frequent consumption of soft, processed foods [13]. Nevertheless, research on the oral health status of children with autism has yielded equivocal findings. While a substantial proportion of research indicates that children with ASD exhibit higher caries prevalence and poorer oral hygiene compared to the control groups, some studies have reported contrasting findings. In particular, these studies have indicated that children with ASD may demonstrate less dental disease, as measured by the sum of decayed, missing and filled adult or primary teeth (DMFT/dmft) [14,15,16,17,18,19,20]. Data syntheses in the form of meta-analyses demonstrate that children diagnosed with ASD exhibit significantly suboptimal hygiene practices when compared to their healthy peers [21,22,23]. The underlying causes of this phenomenon are not yet fully elucidated and may be multifaceted. Higher DMFT/dmft scores may also be attributed to parents focusing on other health and behavioral issues to a greater extent than oral health. Alternatively, this may be the result of a lack of education surrounding successful behavioral management strategies that are available at pediatric dental clinics [24]. Furthermore, children diagnosed with ASD frequently necessitate long-term medication or supplements, which are more readily administered in the form of syrups. The use of oral formulations, which are often sweetened, has been demonstrated to increase the risk of tooth decay [25,26,27,28]. Moreover, the presence of food rejection and a restricted diet was found to be associated with an elevated prevalence of malocclusion and modified Community Periodontal Index scores in children diagnosed with ASD [29]. Du et al. (2019) demonstrated in a study of considerable statistical significance that children diagnosed with ASD are more likely to require parental assistance with toothbrushing. Furthermore, the study revealed that children diagnosed with ASD had less frequent toothbrushing habits and used toothpaste less frequently [30]. The results of the studies demonstrated a statistically significant association between the presence of ASD and diminished oral health-related quality of life in the study population. This finding was observed both among the children themselves and their families [31,32]. Therefore, this study was conducted with the objective of evaluating the oral hygiene status of children with ASD. The hypothesis guiding this study was that children with ASD would demonstrate a higher prevalence of dental cavities and oral hygiene problems.

## 2. Materials and Methods

### 2.1. Study Design

This study was conducted using a cross-sectional design, with data collected between June 2019 and January 2022. The data were then analyzed in order to evaluate the oral health status of children diagnosed with autism spectrum disorder. Prior to participation, written informed consent was obtained from all parents or legal guardians of the children included in the study. The study received formal review and approval from the Bioethics Committee of the Silesian Medical University, with number PCN/CBN/0022/KB1/82/1/19/21. The study complied with the Strengthening the reporting of observational studies in epidemiology (STROBE) statement.

### 2.2. Setting

The medical experiment was conducted at the Persevere Developmental Support Centre (CWR Persevere Niepubliczna Specjalistyczna Poradnia Psychologiczno-Pedagogiczna, 56 Kępowa Street, 40-583 Katowice, Poland). CWR Persevere is a center specializing in the provision of care for individuals diagnosed with autism and their families. CWR Persevere provides care for autistic individuals of various age groups, offering diagnostic services and therapeutic interventions throughout the developmental stage. Individuals diagnosed with autism often exhibit communication impairments and aberrant behavioral tendencies, characterized by an obsessive need to maintain a consistent environment. The integration of the dental examination into the structure of the Persevere CWR has been shown to result in a significant reduction in stress levels among the patient cohort studied.

### 2.3. Study Population

A total of 148 patients were enrolled in the study: including 74 children (boys, girls) diagnosed with autism spectrum disorder, without known confounders (such as mental disability, epilepsy, genetically determined syndromes) aged between 3 and 12 years, and 74 of their peers of both sexes without autistic traits, who formed the control group. Recruitment of healthy children from the control group took place by displaying posters in primary schools and kindergartens in the Silesian Province. Parents and legal guardians voluntarily enrolled their children in the study by signing appropriate consents. Neither the material status nor the educational level of the carers was taken into account.

The inclusion criteria for the study group were as follows:

- Voluntary consent of parent/legal guardian to participate in the study.
- Agefrom 3 to 12 years.
- Diagnosedby a psychiatrist according to the ICD10 classification of child autism (AD) or atypical autism (AA) or Asperger syndrome (AS).
- Noidentified chronic disease potentially affecting nutritional status.

Exclusion criteria were as follows:

- Patientsbelow the age of 3 years and above the age of 12 years.
- Patientsdiagnosed with syndromic autism and additional diagnoses of mental retardation, epilepsy, congenital syndrome, or genetic syndrome.

### 2.4. Data Collection

Prior to the visit, parents were provided with proprietary child adaptation videos demonstrating the step-by-step procedures that would be performed as part of the study. The dental examination was conducted within the confines of a dental office setting, under artificial lighting, using basic diagnostic tools such as a mirror, probe, and spatula. The data obtained were then entered into the examination card. The methodology and scope of the examination were adapted to the age of the child and their level of cooperation. The dental status was meticulously analyzed. The deciduous and permanent teeth marked on the diagram were assessed in terms of the timeliness of their eruption, loss (missing teeth), size, shape, and number. Each tooth was meticulously assessed for foci of primary caries, secondary caries, fillings, possible lesions of non-carious origin, and trauma. The subsequent data analysis led to the calculation of caries dmft and DMFT indexes. The DMFS index was not included in this study as the primary objective was to assess overall caries prevalence rather than specific surface level involvement. The oral hygiene of the subjects was also examined. During the examination, the child was instructed to rinse their mouth with a dissolved GUM Red-Cote plaque staining tablet. The presence of dental deposits, manifesting as plaque or calculus, was made evident through the application of this stain. This method was employed to enhance motivation during the examination. The children were shown the plaque staining and advised to brush their teeth to remove the deposits. Depending on the individual child’s needs, oral hygiene instructions were provided, and treatment or extended diagnosis was recommended. The staining exhibited enabled the determination of the Green and Vermillion Oral Hygiene Index (OHI) and Sulcus Bleeding Index (SBI).

Gingival bleeding was assessed as present (+) or absent (−) after gentle probing of the gingival groove. Assessments were made on the following surfaces: In quadrants 1 and 3—on the buccal and labial surfaces, and in quadrants 2 and 4—on the lingual and palatal surfaces. Spaces in the areas of missing teeth, behind the last teeth and between the upper and lower central incisors were not considered. Each space assessed was given a binary score: 0—no bleeding, 1—presence of bleeding. Calculation of the SBI index: SBI (%) = (Number of bleeding spaces/Number of total spaces assessed) × 100%. Interpretation of SBI results:

≥50%—severe and generalized periodontitis;
30–49%—generalized, moderate periodontitis;
11–29%—localized, mild periodontitis;
<10%—clinically healthy periodontium.

This index allowed us to assess the degree of gingivitis and periodontal health in the study group of children with ASD and the control group

### 2.5. Statistical Analysis

Statistical analysis of the results was performed using STATISTICA v.12 and Excel software. The result of the statistical analysis was considered to be statistically significant if the obtained significance level *p* was less than or equal to 0.05.

Quantitative values were described by giving the mean (X), standard deviation (SD), median (Me), and quartile range (lower quartile Q1; upper quartile Q3) determined from the empirical distribution with interpolation. The Shapiro–Wilk test was used to check the conformity of the analyzed values with a normal distribution.

The Mann–Whitney U test was employed to assess the significance of intergroup differences in the analyzed values, given the rejection of the hypothesis of normality of distributions as indicated by the results of the Shapiro–Wilk test. Nominal and ordinal variables were presented as count (*n*) and frequency (%) distributions. The significance of differences in the distributions of nominal variables was tested using Fisher’s two-sided exact test, and of ordinal variables using the Mann–Whitney U test. For the significant differences obtained in the statistical analyses, the following were used as measures of effect size:

● ϕ—Yule index for Fisher’s exact test (threshold values: 0.10 weak effect, 0.30 moderate effect, 0.50 strong effect.

Finally, the rG was measured using Glass’s rank biserial correlation coefficient for the Mann–Whitney U test, with thresholds of 0.10 indicating a weak effect, 0.30 indicating a moderate effect, 0.50 indicating a strong effect, and 0.70 indicating a very strong effect.

## 3. Results

### 3.1. Demographic Characteristics

A total of 148 Caucasian children of both sexes, aged between 3 and 12 years, were enrolled in the study. Half of these children, 74 in total, were diagnosed by a neurologist and a psychiatrist, according to the International Classification of Diseases, 11th Revision (ICD-11), with autism spectrum disorders (ASD) without comorbidities of the nervous system, in particular without intellectual disability (II ≥ 70), epilepsy, or cerebral palsy. The group is predominantly male, with a ratio of 58 to 16 girls. The control group was also selected in a similar manner, consisting of 53 boys and 21 girls.

Initial analyses were conducted to verify whether the gender distribution and age were similar in the compared groups and between the sexes. To this end, chi-square tests of independence were performed to verify whether the gender distribution was comparable in the ASD and control groups. Additionally, a Mann–Whitney U test was conducted to verify that there were no differences in age between the groups, also by gender subgroups. The analysis with the chi-square test revealed no statistically significant differences, indicating that the gender distribution in both groups is similar.

However, a statistically significant Mann–Whitney U-test result indicates an age difference between female and male children in the autism spectrum group (*p* < 0.001). Specifically, the boys in the study were older than the girls. In contrast, no statistically significant differences in age were observed between girls and boys in the control group, although the age of the boys in this group was also greater. Overall, the two groups did not differ statistically significantly in age or by gender subgroups. Consequently, the two groups were found to be homogeneous in terms of size, age, and gender (Table 1).

### 3.2. Caries Indexes

**Figure 1:**
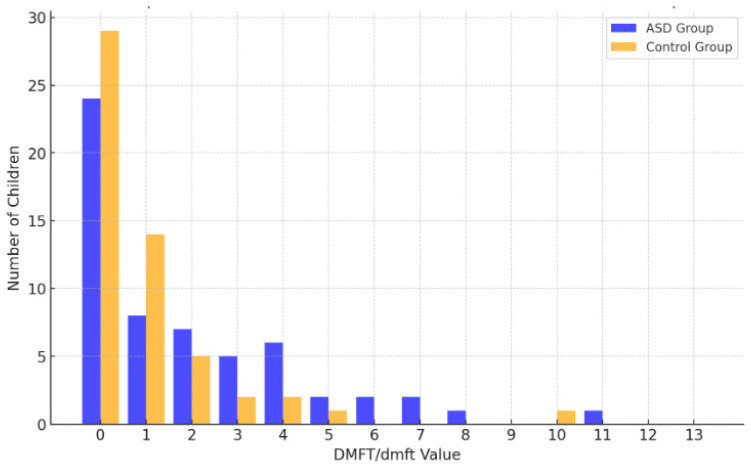
Comparison of DMFT/dmft values in children diagnosed with ASD vs. control group.

The analysis revealed a statistically significant (*p* < 0.05) disparity in DMFT values between the group with ASD and the male control group. The difference was characterized by an average effect size. Boys diagnosed with ASD exhibited higher values for both the DMFT index and dmft index in comparison to the subjects comprising the control group. Within the group of boys with ASD, 19 (32.76%) had DMFT/dmft values at or above 5. In contrast, such values were observed in only five (9.43%) boys in the control group. No boy in the control group had a DMFT value above five, while sixboys in the ASD group did (10.34% of the group) (Figure 1). The remaining differences in the analyzed values were statistically insignificant (Table 2).

### 3.3. OHI and SBI

The Mann–Whitney U test was employed to ascertain whether there were any differences between the group with ASD and the control group in OHI and SBI values. The analysis was performed separately for the female gender, male gender, and total groups. The results of this test showed statistically significant intergroup differences (*p* < 0.001) for the boys and total groups for both OHI and SBI values. The measure expressed by Glass’s coefficient (rG) for both indices indicates a strong and very strong interaction effect of the differences present. The SBI values indicate a 2-fold poorer level of hygiene maintenance in boys on the autism spectrum compared to their peers. In the case of girls diagnosed with ASD, the values of the indicators are elevated in comparison to the control group; however, this difference did not attain statistical significance (Table 3).

### 3.4. Frequency of Tooth Brushing

As demonstrated in Table 4, the distribution of the number of daily tooth brushing occasions by the children in the study is summarized. A statistically significant intergroup difference was recorded for boys (*p* < 0.01) and in the overall analysis (*p* < 0.05). The observed differences were characterized by a weak interaction effect. Boys diagnosed with ASD, in addition to children in the overall ASD group, exhibited a lower frequency of diurnal tooth brushing.

### 3.5. Frequency of Dental Visits

**Figure 2:**
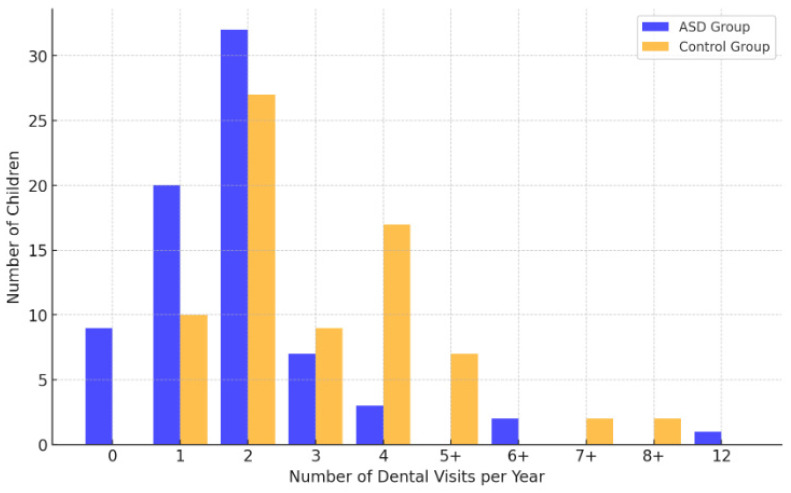
Comparison of frequency of annual dental visits for ASD and control group.

Table 5 presents the distribution of the number of visits to the dentist per year for the children in the study. The results of the intergroup analyses, categorized by gender as well as overall, revealed statistically significant differences with an average (boys, *p* < 0.01) and a very strong interaction effect (girls and overall, *p* < 0.001). Children diagnosed with ASD attended the dentist less frequently during the year. It is noteworthy that no child in the control group was absent from at least one annual check-up. Within the ASD group, nine children were identified as non-attenders, accounting for 12.16% of the group. As illustrated in Figure 2, there is a clear distinction in the frequency of annual dental visits between the ASD and Control groups.

### 3.6. Sealing and Varnishing

In the final stage of the study, the correlation of varnishing and varnishing with DMFT and dmft abnormalities in the ASD and control groups was tested. To this end, a cross-tabulation was conducted in conjunction with two-sided Fisher’s exact tests (Table 6).

The analyses performed yielded no significant results in any case. These findings suggest that, irrespective of the presence or absence of dmft or DMFT abnormalities, there was no increased prevalence of varnishing or sealing in any of the study groups (ASD, control).

## 4. Discussion

The findings of the study lend support to the hypothesis, demonstrating a significantly higher prevalence of dental decay and poorer oral hygiene status in children with ASD compared to the control group. The study demonstrated that children diagnosed with ASD exhibited suboptimal oral hygiene practices when compared to their healthy peers. Boys with ASD exhibited significantly higher DMFT values, suggesting a heightened vulnerability to caries. Furthermore, the analysis of OHI and SBI revealed significantly higher values in boys with ASD, suggesting greater plaque accumulation and a higher frequency of gingival inflammation. Furthermore, children with ASD exhibited a lower frequency of tooth brushing and dental visits in comparison to the control group. Furthermore, it was observed that prophylactic treatments, such as sealants and varnishing, were not utilized more frequently in children with ASD, irrespective of the presence of carious lesions. A substantial body of research, conducted by numerous authors, has corroborated the findings of the present study. The majority of these studies have demonstrated that children diagnosed with ASD exhibit suboptimal levels of personal hygiene and experience worse dental health compared to their healthy counterparts [24,31,33,34,35,36]. Despite these results, El Khatib et al. demonstrated no difference in the prevalence of caries in the deciduous and permanent dentition between children with ASD and healthy children [24]. Nevertheless, there are studies that present findings thatare quite different. In their study, Du et al. demonstrated that children with ASD had a significantly lower dmft/DMFT ratio, and that their gingival condition was better than that of children without an autism spectrum disorder [37]. Loo et al. obtained analogous results; however, it is imperative to consider that this study was conducted on a significantly older cohort of children [38]. A study by Blomqvist et al. found that adult patients diagnosed with ASD exhibited diminished stimulated saliva secretion in comparison to healthy patients. These findings were independent of medication taken. The same study also demonstrated that patients diagnosed with ASD were more prone to forget dental appointments [39]. Moreover, the study by Bhandry et al. revealed that children diagnosed with ASD exhibited lower levels of salivary pH and buffering capacity in comparison with their healthy siblings [35]. It is evident that oral health-related outcomes can be enhanced through the implementation of risk assessment and prevention strategies during early dental visits. These visits, undertaken in the initial phases of primary tooth development, have been shown to minimize the necessity for subsequent treatment and the use of general anesthesia. The establishment of a dental home in early childhood is of paramount importance, and the regular attendance of children with ASD at dental appointments is crucial in reducing the demand for extensive treatment under general anesthesia [16,40,41,42]. It is imperative to acknowledge the challenges faced by children with ASD in maintaining independent oral hygiene, which is compounded by sensory sensitivities, motor challenges, and behavioral resistance. Consequently, parental or caregiver involvement assumes a pivotal role. Research has underscored that regular supervision, assistance, and structured routines result in substantial improvements in oral health outcomes for children with ASD. Caregivers must be equipped with knowledge regarding adaptive brushing techniques, specialized oral care tools, and behaviour management strategies to ensure efficacious hygiene practices. It is recommended that a range of methods of adaptation for children with ASD be implemented in medical facilities. Research has demonstrated the efficacy of dental adaptation strategies, particularly video-modeling and sensory-adapted environments, in enhancing oral health outcomes and reducing anxiety in children with ASD [43,44]. The findings of the present study indicate a necessity for further information to be imparted to parents by pediatricians and dentists regarding the significance of oral health promotion in children diagnosed with ASD. It is recommended that pediatricians encourage parents to seek dental care for periodic dental recalls and reassure them regarding the necessity and safety of topical fluoride exposure. The promotion of positive relationships between pediatric dentists and young people, as well as the reduction in treatment requirements and negative experiences, is crucial. This can be achieved by ensuring that children with ASD receive regular dental care and are familiarized with the need for and safety of topical fluoride exposure. The use of fluoride-containing products such as toothpaste and in-office fluoride varnish is highly recommended for all young people with ASD. Given that oral hygiene is the primary risk factor for the development of dental caries, frequent dental hygiene recalls are deemed necessary. The utilization of pit and fissure sealants is strongly advocated to prevent the development of dental caries in permanent teeth [36]. In view of the elevated risk of dental caries in children diagnosed with ASD, remineralization strategies are of crucial importance in preventing the progression of the disease and restoring enamel integrity (restitutio ad integrum). Recent studies, including those by Scribante et al., have demonstrated the efficacy of biomimetic hydroxyapatite-based remineralization systems in reducing caries susceptibility. The incorporation of hydroxyapatite nanoparticles into routine dental care has been demonstrated to promote non-invasive remineralization and enhance enamel resistance to acid attacks. This is due to the fact that these nanoparticles mimic the natural composition of enamel [45]. Furthermore, the integration of advanced diagnostic tools such as Diagnodent and Diagnocam facilitates the early detection of demineralization, thereby allowing for the timely intervention of remineralizing agents. The results of recent research suggest that the integration of these diagnostic and treatment strategies into routine dental care can significantly improve oral health outcomes in children with ASD, who may struggle with traditional preventive measures [46].

It is important to acknowledge the limitations of the study. A significant constraint is its single-center design, which considerably restricts the study population. The study population of 74 patients with ASD is also a limitation due to the small number of patients included. Another major limitation is the omission of dietary habits, which were not assessed via a questionnaire. Additionally, there was no comprehensive analysis of the impact of behavioral therapy and health education. Furthermore, the results suggest that the poorer health status of children with ASD may be related to less access to doctors specializing in treating children on the autism spectrum. A salient factor that merits consideration is the statistically significant age disparity between boys and girls within the ASD group, with boys exhibiting an average age of 2 years and 9 months more than their female counterparts. Given that caries prevalence typically rises with age due to protracted exposure to cariogenic factors, the observed variations in oral health outcomes may be partly influenced by age rather than gender alone. It is conceivable that older children may have had more cumulative exposure to dietary sugars, less parental supervision over oral hygiene, and increased risk of plaque accumulation, all of which contribute to higher caries prevalence. Therefore, the differences in DMFT/dmft scores between boys and girls with ASD may reflect an age-related trend rather than a true gender disparity. A significant limitation of this study is the absence of data regarding the severity of autism, which may have a crucial impact on oral health outcomes. An additional limitation is also the lack of data on the economic status of parents of children with ASD. Such data could allow important correlations to be noted. Future studies should take this factor into account to eliminate the bias associated with financial and educational differences between parents of children with ASD. Autism is a heterogeneous condition, and it remains unclear whether the observed gender differences in oral health are indicative of true gender disparities or whether they reflect variations in autism severity, sensory sensitivities (e.g., oral aversion), or behavioral challenges such as resistance to oral care routines. While the current study has identified significant disparities in oral health between male and female ASD subjects, it is not possible to exclude the possibility that these disparities are driven by differences in symptom severity rather than gender alone. Future research should therefore stratify participants by ASD severity in order to determine its independent impact on oral health disparities. However, it is important to note that other authors have reached divergent conclusions regarding the relationship between ASD severity and oral hygiene, as well as dental caries indices [39,47].

## 5. Conclusions

This study highlights significant oral health disparities, particularly among boys with ASD, who exhibited poorer oral hygiene, higher caries prevalence, and reduced preventive care compared to their neurotypical counterparts. While differences in oral health indicators among girls were observed, they did not reach statistical significance. These findings underscore the necessity for enhanced oral health interventions, incorporating specialized dental care approaches, augmented parental education, and targeted preventive strategies. Regular dental visits and early intervention are pivotal in mitigating the oral health risks associated with ASD. Future research should explore behavioral adaptation strategies and the impact of dietary habits on oral health in children with ASD. Addressing these challenges requires a multidisciplinary approach involving pediatricians, dentists, and caregivers to ensure comprehensive and effective oral health care for children with ASD.

## Figures and Tables

**Table 1:** Characteristics of the study groups.

	Sex	ASD Group (*n* = 74; 100%)	Control Group (*n* = 74; 100%)	Test of Difference Between Groups
Gender distribution	Female	16 (21.62%)	21 (28.38%)	NS (*p* = 0.45)/*
	Male	58 (78.38%)	53 (71.62%)	
Age (years) Mean ± SD Me [Q1; Q3]	Female	6.9 ± 1.7 7.0 (6.0; 8.0)	7.6 ± 2.4 6.0 (6.0; 10.0)	NS (*p* = 0.66)/#
	Male	9.6 ± 2.0 10.0 (8.0; 11.0)	8.8 ± 2.6 9.0 (7.0; 12.0)	NS (*p* = 0.17)/#
	Group gender difference test	*p* < 0.001/# (ES) rG = 0.66	NS (*p* = 0.054)/#	---
	Total	9.0 ± 2.2 9.0 (7.0; 11.0)	8.5 ± 2.6 8.0 (6.0; 11.0)	NS (*p* = 0.18)/#

SD—standard deviation; Me—median; Q1—lower quartile; Q3—upper quartile. Note: Significance of differences was tested using the CHI2 test with Yates correction (denoted by /*) or the Mann–Whitney U test (denoted by /#); the Glass rank biserial correlation coefficient was used as a measure of ES effect size.

**Table 2:** Gender distributions of DMFT and dmft caries index values in the ASD and control groups.

	DMFT		Dmft	
	Females			
DMFT/dmft value	ASD group *n* = 16 (100%)	Control group *n* = 21 (100%)	ASD group *n* = 16 (100%)	Control group *n* = 21 (100%)
0	11 (68.75%)	11 (52.38)	5 (31.25%)	6 (28.57%)
1	2 (12.50%)	4 (19.05%)	2 (12.50%)	4 (19.05%)
2	0	2 (9.52%)	1 (6.25%)	5 (23.81%)
3	1 (6.25%)	1 (4.76%)	3 (18.75%)	4 (19.05%)
4	2 (12.50%)	3 (14.29%)	1 (6.25%)	0
5	0	0	0	1 (4.76%)
6	0	0	1 (6.25%)	0
7	0	0	0	0
8	0	0	2 (12.50%)	0
Intergroup difference test	NS (*p* = 0.46)		NS (*p* = 0.40)	
	Males			
DMFT/dmft value	ASD group *n* = 58 (100%)	Control group *n* = 53 (100%)	ASD group *n* = 58 (100%)	Control group *n* = 53 (100%)
0	24 (41.38%)	29 (54.72%)	25 (43.10%)	16 (30.16%)
1	8 (13.79%)	14 (26.42%)	5 (8.62%)	14 (26.42%)
2	7 (12.07%)	5 (9.43%)	7 (12.07%)	12 (22.64%)
3	5 (8.62%)	2 (3.77%)	5 (8.62%)	2 (3.77%)
4	6 (10.34%)	2 (3.77%)	4 (6.90%)	5 (9.43%)
5	2 (3.45%)	1 (1.89%)	1 (1.72%)	2 (3.77%)
6	2 (3.45%)	0	6 (10.34%)	1 (1.89%)
7	2 (3.45%)	0	1 (1.72%)	0
8	1 (1.72%)	0	0	0
9	0	0	2 (3.45%)	0
10	0	0	0	1 (1.89%)
11	1 (1.72%)	0	1 (1.72%)	0
12	0	0	0	0
13	0	0	1 (1.72%)	0
Intergroup difference test	*p* < 0.05; (ES) rG = 0.25		NS (*p* = 0.76)	
	Total			
DMFT/dmft value	ASD group *n* = 74 (100%)	Control group *n* = 74 (100%)	ASD group *n* = 74 (100%)	Control group *n* = 74 (100%)
0	35 (47.30%)	40 (54.05%)	30 (40.54%)	22 (29.73%)
1	10 (13.51%)	18 (24.32%)	7 (9.46%)	18 (24.32%)
2	7 (9.46%)	7 (9.46%)	8 (10.81%)	17 (22.97%)
3	6 (8.11%)	3 (4.05%)	8 (10.81%)	6 (8.11%)
4	8 (10.81%)	5 (6.76%)	5 (6.76%)	5 (6.76%)
5	2 (2.70%)	1 (1.35%)	1 (1.35%)	3 (4.05%)
6	2 (2.70%)	0	7 (9.46%)	1 (1.35%)
7	2 (2.70%)	0	1 (1.35%)	0
8	1 (1.35%)	0	2 (2.70%)	1 (1.35%)
9	0	0	3 (4.05%)	0
10	0	0	0	1 (1.35%)
11	1 (1.35%)	0	1 (1.35%)	0
12	0	0	0	0
13	0	0	1 (1.35%)	0
Intergroup difference test	NS (*p* = 0.071)		NS (*p* = 0.53)	

Note: Significance of differences was tested using the Mann–Whitney U test; the Glass rank bivariate correlation coefficient was used as a measure of ES effect size.

**Table 3:** OHI and SBI values in the ASD and control groups by gender.

Index	Gender	ASD Group	Control Group	Test of Difference Between Groups
OHI Mean ±SD Me [Q1; Q3]	Female	2.26 ± 1.28 2.80 [2.00; 3.00]	1.90 ± 1.15 2.00 [1.00; 3.00]	NS (*p* = 0.32)
	Male	2.57 ± 0.66 2.80 [2.35; 3.00]	2.08 ± 0.81 2.00 [1.50; 2.60]	*p* < 0.001; (ES) rG = 0.46
	Group gender difference test	NS (*p* = 0.59)	NS (*p* = 0.62)	===
	Total	2.50 ± 0.83 2.80 [2.30; 3.00]	2.03 ± 0.91 2.00 [1.50; 2.80]	*p* < 0.001; (ES) rG = 0.38
SBI Mean ± SD Me [Q1; Q3]	Female	27.2 ± 12.5 25.0 [18.8; 35.0]	22.3 ± 11.5 20.0 [15.0; 28.0]	NS (*p* = 0.20)
	Male	30.5 ± 9.6 33.0 [25.0; 35.0]	17.6 ± 6.4 20.0 [10.0; 20.0]	*p* < 0.001; (ES) rG = 0.71
	Group gender difference test	NS (*p* = 0.36)	NS (*p* = 0.21)	===
	Ogółem	29.8 ± 10.3 31.0 [25.0; 35.0]	18.9 ± 8.4 20.0 [11.3; 20.0]	*p* < 0.001; (ES) rG = 0.59

SD—standard deviation; Me—median; Q1—lower quartile; Q3—upper quartile. Note: Significance of differences was tested using the CHI2 test with Yates correction (labelled /*) or the Mann–Whitney U test (oz; labelled /#); the Glass rank bivariate correlation coefficient was reported as a measure of ES effect size.

**Table 4:** Gender distributions of the number of daily teeth brushings by children of the ASD and control groups.

	Females	
Number of daily teeth brushings	ASD group *n* = 16 (100%)	Control group *n* = 21 (100%)
1	2 (12.50%)	3 (14.29%)
2	10 (62.50%)	11 (52.38%)
3	0	4 (19.05%)
4	3 (18.75%)	3 (14.29%)
5	1 (6.25%)	0
U Mann–Whitney test	NS (*p* = 1.00)	
	Males	
Number of daily teeth brushings	ASD group *n* = 58 (100%)	Control group *n* = 53 (100%)
1	10 (17.24%)	7 (13.21%)
2	40 (68.97%)	25 (47.17%)
3	7 (12.07%)	12 (22.64%)
4	1 (1.72%)	7 (13.21%)
5	0	2 (3.77%)
U Mann–Whitney test	*p* < 0.01; (ES) rG = 0.27	
	Total	
Number of daily tooth brushing	ASD group *n* = 74 (100%)	Control group *n* = 74 (100%)
1	12 (16.22%)	10 (13.51%)
2	50 (67.57%)	36 (48.65%)
3	7 (9.46%)	16 (21.62%)
4	4 (5.41%)	10 (13.51%)
5	1 (1.35%)	2 (2.70%)
U Mann–Whitney test	*p* < 0.05; (ES) rG = 0.20	

Note: Glass’s rank bivariate correlation coefficient (rG) is given as a measure of ES effect size.

**Table 5:** Gender distributions of the number of annual visits to the dentist by children of the ASD and control groups.

	Females	
Number of visits in 12 months	ASD group *n* = 16 (100%)	Control group *n* = 21 (100%)
0	6 (37.50%)	0
1	4 (25.00%)	2 (9.52%)
2	6 (37.50%)	8 (38.10%)
3	0	1 (4.76%)
4	0	8 (38.10%)
5	0	0
6	0	0
7	0	1 (4.76%)
8	0	1 (4.76%)
12	0	0
U Mann–Whitney test	*p* < 0.001; (ES) rG = 0.76	
	Males	
Number of visits in 12 months	ASD group *n* = 58 (100%)	Control group *n* = 53 (100%)
0	3 (5.17%)	0
1	16 (27.59%)	8 (15.09%)
2	26 (44.83%)	19 (35.85%)
3	7 (12.07%)	8 (15.09%)
4	3 (5.17%)	9 (16.98%)
5	0	7 (13.21%)
6	2 (3.45%)	0
7	0	1 (1.89%)
8	0	1 (1.89%)
12	1 (1.72%)	0
U Mann–Whitney test	*p* < 0.01; (ES) rG = 0.34	
	Total	
Number of visits in 12 months	ASD group *n* = 74 (100%)	Control group *n* = 74 (100%)
0	9 (12.16%)	0
1	20 (27.03%)	10 (13.51%)
2	32 (43.24%)	27 (36.49%)
3	7 (9.46%)	9 (12.16%)
4	3 (4.05%)	17 (22.97%)
5	0	7 (9.46%)
6	2 (2.70%)	0
7	0	2 (2.70%)
8	0	2 (2.70%)
12	1 (1.35%)	0
U Mann–Whitney test	*p* < 0.001; (ES) rG = 0.44	

Note: Glass’s rank bivariate correlation coefficient (rG) is given as a measure of ES effect size.

**Table 6:** Correlation of sealing and varnishing with DMFT and dmft abnormalities in ASD and control groups.

	ASD Group		Control Group	
	DMFT irregularities			
Varnishing	Present (*n* = 39; 100%)	Not present (*n* = 35; 100%)	Present (*n* = 34; 100%)	Not present (*n* = 40; 100%)
No	34 (87.18%)	30 (85.71%)	28 (82.35%)	34 (85.00%)
Yes	5 (12.82%)	5 (14.29%)	6 (17.65%)	6 (15.00%)
Fisher’s two-sided exact test	NS (*p* = 1.00)		NS (*p* = 0.76)	
	DMFT irregularities			
Sealing	Present (*n* = 39; 100%)	Not present (*n* = 35; 100%)	Present (*n* = 34; 100%)	Not present (*n* = 40; 100%)
No	26 (66.67%)	27 (77.14%)	15 (44.12%)	16 (40.00%)
Yes	13 (33.33%)	8 (22.86%)	19 (55.88%)	24 (60.00%)
Fisher’s two-sided exact test	NS (*p* = 0.44)		NS (*p* = 0.81)	
	dmft irregularities			
Varnishing	Present (*n* = 44; 100%)	Not present (*n* = 30; 100%)	Present (*n* = 52; 100%)	Not present (*n* = 22; 100%)
No	38 (86.36%)	26 (86.67%)	43 (82.69%)	19 (86.36%)
Yes	6 (13.64%)	4 (13,33%)	9 (17.31%)	3 (13.64%)
Fisher’s two-sided exact test	NS (*p* = 1.00)		NS (*p* = 1.00)	
	dmft irregularities			
Sealing	Present (*n* = 44; 100%)	Not present (*n* = 30; 100%)	Present (*n* = 52; 100%)	Not present (*n* = 22; 100%)
No	33 (75.00%)	20 (66.67%)	20 (38.46%)	11 (50.00%)
Yes	11 (25.00%)	10 (33.33%)	32 (61.54%)	11 (50.00%)
Fisher’s two-sided exact test	NS (*p* = 0.45)		NS (*p* = 0.44)	

## Data Availability

The raw data supporting the conclusions in this article will be made available by the authors on request.

## References

[1] Gosling C.J., Cartigny A., Mellier B.C., Solanes A., Radua J., Delorme R. (2022). Efficacy of Psychosocial Interventions for Autism Spectrum Disorder: An Umbrella Review. Mol. Psychiatry.

[2] Frye R.E. (2022). A Personalized Multidisciplinary Approach to Evaluating and Treating Autism Spectrum Disorder. J. Pers. Med..

[3] Hirota T., King B.H. (2023). Autism Spectrum Disorder: A Review. JAMA.

[4] Bernath B., Kanji Z. (2021). Exploring Barriers to Oral Health Care Experienced by Individuals Living with Autism Spectrum Disorder. Can. J. Dent. Hyg..

[5] Farooq M.S., Tehseen R., Sabir M., Atal Z. (2023). Detection of Autism Spectrum Disorder (ASD) in Children and Adults Using Machine Learning. Sci. Rep..

[6] Park M.N., Moulton E.E., Laugeson E.A. (2023). Parent-Assisted Social Skills Training for Children With Autism Spectrum Disorder: PEERS for Preschoolers. Focus. Autism Other Dev. Disabl..

[7] Carli E., Pasini M., Pardossi F., Capotosti I., Narzisi A., Lardani L. (2022). Oral Health Preventive Program in Patients with Autism Spectrum Disorder. Children.

[8] Rice C.E., Baio J., Van Naarden Braun K., Doernberg N., Meaney F.J., Kirby R.S. (2007). ADDM Network A Public Health Collaboration for the Surveillance of Autism Spectrum Disorders. Paediatr. Perinat. Epidemiol..

[9] Ferrazzano G.F., Salerno C., Bravaccio C., Ingenito A., Sangianantoni G., Cantile T. (2021). Autism spectrum disorders and oral health status: Review of the literature. Eur. J. Paediatr. Dent..

[10] De Almeida J.S., Fernandes R.F., Andrade Á.C.B., Almeida B.D.C., Amorim A.N.D.S., Lustosa J.H.D.C.M., Mendes R.F., Prado Júnior R.R. (2021). Impact of Dental Treatment on the Oral Health-related Quality of Life of Children and Adolescents with Autism Spectrum Disorder. Spec. Care Dent..

[11] Ferreira M.C., Goursand D., Bendo C.B., Ramos-Jorge M.L., Pordeus I.A., Paiva S.M. (2012). Agreement between Adolescents’ and Their Mothers’ Reports of Oral Health-Related Quality of Life. Braz. Oral Res..

[12] Jokovic A., Locker D., Stephens M., Kenny D., Tompson B., Guyatt G. (2003). Measuring Parental Perceptions of Child Oral Health-related Quality of Life. J. Public Health Dent..

[13] Butera A., Maiorani C., Morandini A., Simonini M., Morittu S., Trombini J., Scribante A. (2022). Evaluation of Children Caries Risk Factors: A Narrative Review of Nutritional Aspects, Oral Hygiene Habits, and Bacterial Alterations. Children.

[14] Gandhi R.P., Klein U. (2014). Autism Spectrum Disorders: An Update on Oral Health Management. J. Evid. Based Dent. Pract..

[15] Zhang Y., Lin L., Liu J., Shi L., Lu J. (2020). Dental Caries Status in Autistic Children: A Meta-Analysis. J. Autism Dev. Disord..

[16] Hasell S., Hussain A., Da Silva K. (2022). The Oral Health Status and Treatment Needs of Pediatric Patients Living with Autism Spectrum Disorder: A Retrospective Study. Dent. J..

[17] Rouleau T., Harrington A., Brennan M., Hammond F., Hirsch M., Nussbaum M., Bockenek W. (2011). Receipt of Dental Care and Barriers Encountered by Persons with Disabilities. Spec. Care Dent..

[18] Namal N., Vehit H., Koksal S. (2007). Do Autistic Children Have Higher Levels of Caries? A Cross-Sectional Study in Turkish Children. J. Indian Soc. Pedod. Prev. Dent..

[19] Jaber M.A. (2011). Dental Caries Experience, Oral Health Status and Treatment Needs of Dental Patients with Autism. J. Appl. Oral Sci..

[20] Lai B., Milano M., Roberts M.W., Hooper S.R. (2012). Unmet Dental Needs and Barriers to Dental Care Among Children with Autism Spectrum Disorders. J. Autism Dev. Disord..

[21] Da Silva S.N., Gimenez T., Souza R.C., Mello-Moura A.C.V., Raggio D.P., Morimoto S., Lara J.S., Soares G.C., Tedesco T.K. (2017). Oral Health Status of Children and Young Adults with Autism Spectrum Disorders: Systematic Review and Meta-analysis. Int. J. Paediatr. Dent..

[22] Corridore D., Zumbo G., Corvino I. (2020). Prevalence of Oral Disease and Treatment Types Proposed Tochildren Affected by Autistic Spectrum Disorder in Pediatric Dentistry: A Systematic Review. Clin. Ter..

[23] Sami W., Ahmad M.S., Shaik R.A., Miraj M., Ahmad S., Molla M.H. (2023). Oral Health Statuses of Children and Young Adults with Autism Spectrum Disorder: An Umbrella Review. J. Clin. Med..

[24] El Khatib A.A., El Tekeya M.M., El Tantawi M.A., Omar T. (2014). Oral Health Status and Behaviours of Children with Autism Spectrum Disorder: A Case–Control Study. Int. J. Paediatr. Dent..

[25] Humaid J.A. (2018). Sweetener Content and Cariogenic Potential of Pediatric Oral Medications: A Literature. Int. J. Health Sci. Res..

[26] Pomarico L., Souza I.P.R., Tura L.F.R. (2005). Sweetened Medicines and Hospitalization: Caries Risk Factors in Children with and without Special Needs. Eur. J. Paediatr. Dent..

[27] Myers S.M., Johnson C.P., The Council on Children with Disabilities (2007). Management of Children with Autism Spectrum Disorders. Am. Acad. Pediatr..

[28] Fallea A., Vetri L., L’Episcopo S., Bartolone M., Zingale M., Di Fatta E., d’Albenzio G., Buono S., Roccella M., Elia M. (2024). Oral Health and Quality of Life in People with Autism Spectrum Disorder. J. Clin. Med..

[29] Leiva-García B., Planells E., Planells Del Pozo P., Molina-López J. (2019). Association Between Feeding Problems and Oral Health Status in Children with Autism Spectrum Disorder. J. Autism Dev. Disord..

[30] Du R.Y., Yiu C.K.Y., King N.M. (2019). Oral Health Behaviours of Preschool Children with Autism Spectrum Disorders and Their Barriers to Dental Care. J. Autism Dev. Disord..

[31] Qiao Y., Shi H., Wang H., Wang M., Chen F. (2020). Oral Health Status of Chinese Children With Autism Spectrum Disorders. Front. Psychiatry.

[32] Da Silva A.C.F., Barbosa T.D.S., Gavião M.B.D. (2023). Parental Perception of the Oral Health-Related Quality of Life of Children and Adolescents with Autism Spectrum Disorder (ASD). Int. J. Environ. Res. Public Health.

[33] DeMattei R., Cuvo A., Maurizio S. (2007). Oral Assessment of Children with an Autism Spectrum Disorder. J. Dent. Hyg..

[34] Mansoor D., Al Halabi M., Khamis A.H., Kowash M. (2018). Oral Health Challenges Facing Dubai Children with Autism Spectrum Disorder at Home and in Accessing Oral Health Care. Eur. J. Paediatr. Dent..

[35] Bhandary S., Hari N. (2017). Salivary Biomarker Levels and Oral Health Status of Children with Autistic Spectrum Disorders: A Comparative Study. Eur. Arch. Paediatr. Dent..

[36] Bagattoni S., Lardani L., D’Alessandro G., Piana G. (2021). Oral Health Status of Italian Children with Autism Spectrum Disorder. Eur. J. Paediatr. Dent..

[37] Du R.Y., Yiu C.K., King N.M., Wong V.C., McGrath C.P. (2015). Oral Health among Preschool Children with Autism Spectrum Disorders: A Case-Control Study. Autism.

[38] Loo C.Y., Graham R.M., Hughes C.V. (2008). The Caries Experience and Behavior of Dental Patients With Autism Spectrum Disorder. J. Am. Dent. Assoc..

[39] Blomqvist M., Bejerot S., Dahllöf G. (2015). A Cross-Sectional Study on Oral Health and Dental Care in Intellectually Able Adults with Autism Spectrum Disorder. BMC Oral Health.

[40] Weninger A., Seebach E., Broz J., Nagle C., Lieffers J., Papagerakis P., Da Silva K. (2022). Risk Indicators and Treatment Needs of Children 2–5 Years of Age Receiving Dental Treatment under General Anesthesia in Saskatchewan. Dent. J..

[41] Nowak A.J., Casamassimo P.S. (2002). The Dental Home: A Primary Care Oral Health Concept. J. Am. Dent. Assoc..

[42] Janik K., Niemczyk W., Peterek R., Rój R., Balicz A., Morawiec T. (2024). Computer-Controlled Local Anaesthesia Delivery Efficacy—A Literature Review. Saudi Dent. J..

[43] Prynda M., Pawlik A.A., Niemczyk W., Wiench R. (2024). Dental Adaptation Strategies for Children with Autism Spectrum Disorder—A Systematic Review of Randomized Trials. J. Clin. Med..

[44] Niemczyk W., Balicz A., Lau K., Morawiec T., Kasperczyk J. (2024). Factors Influencing Peri-Extraction Anxiety: A Cross-Sectional Study. Dent. J..

[45] Butera A., Gallo S., Pascadopoli M., Montasser M.A., Abd El Latief M.H., Modica G.G., Scribante A. (2022). Home Oral Care with Biomimetic Hydroxyapatite vs. Conventional Fluoridated Toothpaste for the Remineralization and Desensitizing of White Spot Lesions: Randomized Clinical Trial. Int. J. Environ. Res. Public Health.

[46] Scribante A., Cosola S., Pascadopoli M., Genovesi A., Battisti R.A., Butera A. (2025). Clinical and Technological Evaluation of the Remineralising Effect of Biomimetic Hydroxyapatite in a Population Aged 6 to 18 Years: A Randomized Clinical Trial. Bioengineering.

[47] Helton J.J., Koetting C., Kronk R., Kong V., Kim Y.S. (2024). Autism Severity, Adverse Childhood Experiences, and Oral Health: A Comparative Study of Adolescents in the United States. J. Autism Dev. Disord..

